# The Economic Impact of Treatment Sequencing in Chronic Lymphocytic Leukemia in Canada Using Venetoclax plus Obinutuzumab

**DOI:** 10.3390/cancers16183182

**Published:** 2024-09-17

**Authors:** Kimberly Guinan, Karine Mathurin, Jean Lachaine, Nancy Paul Roc, Sarah-Jane Bull, Dipti Tankala, Stephane Barakat, Beenish S. Manzoor, Christopher Hillis, Versha Banerji

**Affiliations:** 1 PeriPharm Inc., Montreal, QC H2Y 2H4, Canada; kimberly.guinan@peripharm.com (K.G.); karine.mathurin@peripharm.com (K.M.); 2 Faculty of Pharmacy, University of Montreal, Montreal, QC H3T 1J4, Canada; 3 AbbVie Corporation, Saint-Laurent, QC H4S 1Z1, Canada; nancy.paulroc@abbvie.com (N.P.R.); sarahjane.bull@abbvie.com (S.-J.B.); dipti.tankala@abbvie.com (D.T.); stephane.s.barakat@abbvie.com (S.B.); 4 AbbVie Inc., North Chicago, IL 60064, USA; beenish.manzoor@abbvie.com; 5 Faculty of Health Sciences, McMaster University, Hamilton, ON L8S 4L8, Canada; hillisc@mcmaster.ca; 6 CancerCare Manitoba Research Institute, Winnipeg, MB R3E 0V9, Canada; vbanerji@cancercare.mb.ca; 7 Departments of Internal Medicine and Biochemistry & Medical Genetics, Rady Faculty of Health Sciences, Max Rady College of Medicine, University of Manitoba, Winnipeg, MB R3E 0W2, Canada

**Keywords:** chronic lymphocytic leukemia, BTK inhibitors, treatment sequencing, fixed treatment duration, partitioned survival model

## Abstract

**Simple Summary:**

Bruton tyrosine kinase inhibitors agents are administered continuously until disease progression or unacceptable toxicity, raising concerns about their affordability. Considering the rapidly evolving treatment landscape in chronic lymphocytic leukemia (CLL), ongoing evaluation of CLL treatment sequencing is vital for optimal patient management while ensuring fiscal sustainability. Venetoclax + obinutuzumab (VO) is a fixed-duration treatment (12 months) which has the potential to reduce the cost burden of treating CLL. The aim of this study was to estimate the cumulative cost of different treatment sequences and evaluate the economic impact of introducing treatment sequences with/without VO, from a Canadian health care system perspective. Results highlight that treatment sequences with time-limited therapy VO in first-line resulted in lower costs compared to sequences without VO. Given the expected increase in spending on CLL treatments in Canada, this study indicates a possible strategy to mitigate these rising costs in a publicly funded health care system.

**Abstract:**

Background: Bruton tyrosine kinase inhibitors (BTKis) represent an advancement in chronic lymphocytic leukemia; however, these agents are administered continuously until disease progression or unacceptable toxicity, raising concerns about their affordability. Venetoclax in combination with obinutuzumab (VO) is a fixed-duration (12-month) treatment, approved in Canada in 2020. This study estimated the total cumulative cost of different treatment sequences and evaluated the economic impact of introducing treatment sequences with/without VO, from a Canadian health care system perspective. Methods: A 10-year partitioned survival model was developed, considering key clinical parameters and direct medical costs. Results were stratified by TP53 aberration. Results: Treatment sequences starting with first-line (1L) VO resulted in lower 10-year cumulative costs compared to sequences starting with BTKis administered until disease progression, across both TP53 aberration subgroups. With a maximum of three lines of treatment over a 10-year period, cumulative costs were largely determined by the first two lines of treatment. When comparing sequences with the same 1L treatment, sequences with BTKis in second-line incurred greater costs compared to fixed-duration regimens. Conclusions: Overall, the economic impact of treating all patients with VO led to 10-year cumulative savings of CAD 169,341 and CAD 293,731 per patient, without and with TP53 aberration, respectively. These savings are mainly due to reductions in treatment costs associated with fixed treatment duration.

## 1. Introduction

Chronic lymphocytic leukemia (CLL) is the most common form of adult leukemia in Canada, representing almost half of all leukemia cases [1,2,3,4]. Although there is no cure for CLL, the development and availability of highly effective therapies, including Bruton tyrosine kinase inhibitors (BTKis), have resulted in prolonged survival for these patients [3,5,6]. A substantial proportion of health care spending on CLL is attributed to drug treatment, which poses an economic burden on patients, payers, and society [2,7,8,9,10]. Higher costs are also a consequence of therapy that results in adverse events (AEs), infections, and drug resistance [9,10]. Given that CLL treatments were traditionally agnostic to molecular profiles, patients with adverse molecular features have short remissions, resulting in increased costs of first-line treatment (1L), especially with chemoimmunotherapy [11]. The use of additional lines of therapy is also associated with further decline in quality of life (QoL) and greater economic burden [9]. Additionally, with the evolution of molecular testing, the presence of TP53 aberration is associated with chemoimmunotherapy resistance, while patients with unmutated immunoglobulin heavy-chain variable (IGHV) have shorter remissions [12,13,14,15]. Newer agents, including continuous BTKis (e.g., ibrutinib [IBRU] and acalabrutinib [ACAL]), have demonstrated improved outcomes among CLL patients with these mutations, which has resulted in greater utilization of these therapies in 1L and, hence, greater drug spending on these patients [6,14,16]. 

Though BTKis represent a significant advancement in the management of CLL, their high costs raise concerns about their financial impact and affordability [8,16]. A part of this burden is associated with their use on a continuous basis until disease progression or unacceptable toxicity [8,17]. In a previous study, a model was developed to predict the future prevalence and economic burden of CLL in the era of BTKis in Canada (from 2011 to 2025), which found that the prevalence of CLL is projected to increase almost two-fold and, accordingly, the total annual cost of CLL management is expected to be approximately 16 times greater (rising from approximately CAD 61 to CAD 958 million) by 2025 [8]. The introduction of oral targeted therapies administered for a fixed duration (e.g., venetoclax [V]-based combinations) offers new options for CLL patients with the potential to achieve long-term remission with manageable side effects for a limited period and, most importantly, time off therapy, due the depth of response and marrow clearance [17,18]. From a health care system perspective, a fixed treatment duration has the potential to offer better budget predictability and reduced costs [8,16].

Considering the rapidly evolving treatment landscape in CLL, ongoing evaluation of CLL treatment sequencing is vital to the optimal management of these patients while ensuring fiscal sustainability [16]. Venetoclax (V) in combination with obinutuzumab (VO) is a fixed-duration treatment (12 months) approved in Canada. VO demonstrated significant improvement in progression-free survival (PFS) compared to chemoimmunotherapy (CIT), even four years following treatment cessation [19]. Given its fixed duration of treatment, VO also has the potential to reduce the cost burden of treating CLL. Evaluating the cost implications of VO in various CLL treatment sequences would be beneficial to capture the potential cost savings for the Canadian healthcare system.

The primary objective of this study was to estimate the total cumulative cost per patient of different treatment sequences for adults with CLL, considering 1L and subsequent lines of treatment, over a 10-year time horizon, from a Canadian health care system perspective. A secondary objective was to evaluate the economic impact of introducing treatment sequences with VO as 1L for all CLL patients, compared to sequences without VO in 1L to assess the impact of novel agent fixed-duration therapy. The aim of the study is not to recommend or evaluate clinical protocols. Healthcare professionals should consult Canadian evidence-based guidelines for clinical guidance on CLL treatment [20,21].

## 2. Materials and Methods

### 2.1. Patient Population

The population of interest included all CLL patients requiring frontline therapy. A subgroup analysis was conducted according to TP53 aberration to assess the economic impact within these subgroups of the CLL population.

### 2.2. Model Structure

**Figure 1:**
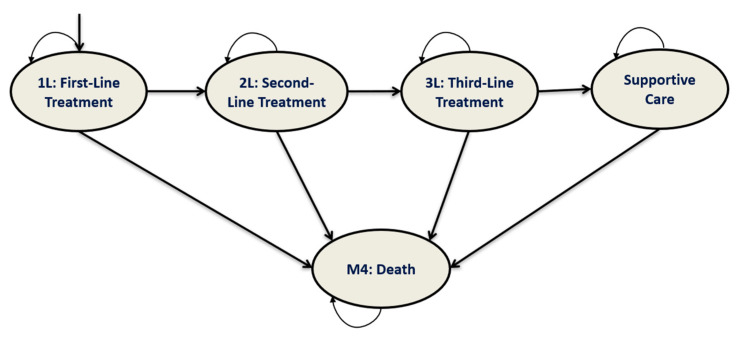
Markov model structure for chronic lymphocytic leukemia (CLL) patients. This health-state transition model comprised 5 health states: first-line treatment (1L), second-line treatment (2L), third-line treatment (3L), supportive care, and death. The model simulates the course of progression of patients with CLL, where all patients enter the model in the 1L health state.

A 10-year partitioned survival model was developed which took into consideration the clinical course of the disease and included the following health states: 1L, second-line treatment (2L), third-line treatment (3L), supportive care, and death (Figure 1) [22].

### 2.3. Simulated Clinical Pathway

Patients enter the model in 1L. If patients fail to respond to 1L, they proceed to 2L. After failure to a 2L, patients proceed to the 3L treatment. If disease progression continues, patients are placed in supportive care (palliative state). Patients in each of the health states could transition to death. Patients could not revert to previous health states.

The probabilities of health-state transitions were estimated based on PFS and overall survival (OS) from pivotal clinical trials (Table 1) [15,19,23,24,25,26,27,28,29,30,31,32,33,34,35,36,37,38]. Trials were selected based on the Canada’s Drug Agency (CDA), formerly known as the Canadian Agency for Drug and Technologies in Health (CADTH), funding algorithm published in May 2021 and supported with more abstracts/publications when available [39]. A PubMed search was performed on June 30, 2022, including pivotal clinical trials (i.e., phase III trials) of key treatments, excluding real-world data. Many new data were also extracted from conference abstracts, which are often not indexed in bibliographical databases such as PubMed. PFS was used to estimate the transition from 1L to 2L, 2L to 3L, and 3L to supportive care. OS was used to determine the transition to death, from all health states. PFS and OS probabilities were converted into 28-day model cycle probabilities using the conversion equations published by Fleurence (2007) [40]. Note that PFS and OS data for 2L and 3L were assumed to be similar, due to the paucity of clinical trial data distinguishing the line of treatment in relapsed/refractory CLL. 

### 2.4. Treatment Sequencing

Treatment sequences (Tables S1 and S2) were defined according to the CDA provisional funding algorithms for CLL published in May 2021 as well as the Alberta clinical guidelines and adapted by CLL clinical experts [35,39,48]. Sequences are also in line with the Canadian evidence-based guidelines for the frontline treatment of CLL [20]. The included treatments are approved and funded therapies in at least one jurisdiction in Canada. Compassionate use was not considered within this model since the objective was to capture the economic impact of various CLL treatment sequences from the public health care system perspective. Note that this research was conducted in 2021/2022 and further changes to the treatment landscape following the study is not captured.

The identification process of treatment sequences was performed by two CLL clinical experts, over two consecutive meetings. The selection process was facilitated by an Excel worksheet (Microsoft Office 365, version 2408), for the selection of 1L, 2L, and 3L treatments. The selected treatment sequences were reviewed and approved by both CLL clinical experts. Note that the total number of treatment sequences was arbitrarily suggested to be limited to 100, of which only 88 were identified.

### 2.5. Cost Data

This economic evaluation was conducted from a Canadian publicly funded health care system perspective. Only direct medical costs relevant to a provincial health care payer were considered, including treatment acquisition costs, administration costs, follow-up and monitoring costs (e.g., laboratory and professional fee costs), cost of tumor lysis syndrome (TLS) prophylaxis, cost of AEs, and cost of supportive care (Table 2 and Tables S3–S12). Cost and resource use data were obtained from various Canadian sources, including published literature, public databases, and clinical experts, to the extent feasible [44,45,49,50,51,52,53,54,55,56,57,58]. All costs were inflated to 2022 Canadian dollars. Discounting was not applied considering that this study is a cost burden analysis, which is modelled similarly to budget impact analyses.

#### 2.5.1. Treatment Acquisition Costs

The unit cost of treatments was obtained from IQVIA Delta PA, as of December 10, 2021. Treatment regimens were obtained from Cancer Care Ontario (CCO) as well as clinical trials [49]. For IV treatments, a body surface area of 1.89 m^2^ and a weight of 76 kg were used to calculate total treatment costs. Drug wastage was not considered. The total treatment costs per 28-day cycle are detailed in Table S3.

#### 2.5.2. Administration Costs

No administration costs were considered for oral therapies, including IBRU, ACAL, and V. For intravenous (IV) regimens, administration costs were estimated based on pharmacist and nurse workloads using information retrieved from CCO, calculated per 28-day treatment cycle [49]. The mean time per occupation was multiplied by their respective median wage, obtained from the Job Bank of Canada (Table 2) [51]. For the pharmacist, only the median annual salary was available; the hourly wage was obtained by assuming 52 weeks of work per year, with an average of 38.5 hours of work per week, as determined by Statistics Canada [52]. The physician supervision cost was also considered and retrieved from the Ontario Schedule of Benefits for Physician Services (Table 2) [50]. The average administration costs per 28-day cycle, per treatment, are detailed in Table S4.

#### 2.5.3. Follow-Up and Monitoring Costs

Routine follow-up and monitoring frequencies per treatment were derived from CCO regimens and included a physician consultation fee, laboratory tests, as well as costs of TLS prophylaxis [49]. 

The physician consultation fee was obtained from the Schedule of Benefits for Physician Services (Table 2) [50]. The total professional fee costs per treatment per 28-day cycle are detailed in Table S5. The unit cost of each laboratory test was retrieved from the Ontario Schedule of Benefits for laboratory services (Table 2) [53]. The total routine costs of laboratory test for each treatment regimen, per 28-day cycle, are detailed in Table S6. In clinical trials, TLS prophylaxis was given to patients who were administered V [6,7,8]; therefore, the cost of TLS prophylaxis was only applied to regimens including V (e.g., VO, V, and V in combination with rituximab [VR]). Costs were assumed to be different for low-risk, moderate-risk, and high-risk patients. The proportions of risk category reported in V clinical trials were used (Table S7). The unit cost of resources for TLS prophylaxis are detailed in Table S8, while the algorithms used to determine the resource use for VO, as well as V/VR, are shown in Tables S9 and S10, respectively. The algorithms were reviewed and approved by clinical experts.

#### 2.5.4. Cost of Adverse Events

The main AEs considered in the model were anemia, neutropenia, febrile neutropenia, thrombocytopenia, bleeding, infection, and atrial fibrillation (Table 2). Only grade 3 or 4 AEs were considered. The rate of AEs for each treatment regimen was obtained from their respective clinical trials. The weighted average cost of AEs per treatment regimen for 1L and 2L/3L are presented in Tables S11 and S12, respectively.

### 2.6. Model Outcomes

The total 10-year cumulative costs per patient, of each treatment sequence, was calculated. Analyses included the stratification of treatment sequences by 1L as well as the first two lines of treatment. In addition, an analysis of the economic impact of treating all CLL patients with treatment sequences starting with VO versus treatment sequences without VO was also performed. The proportion of use of each treatment sequence, for both scenarios, was established by clinical experts in the field of CLL in Canada (Tables S1 and S2). Experts guided their assessment of use according to the funding status and availability of treatments across Canada as well as the Canadian CLL epidemiology and patient stratification (i.e., age, fitness level, IGHV mutation status). All results were stratified by TP53 aberration.

## 3. Results

### 3.1. 10-Year Cumulative Costs

#### 3.1.1. First-Line Treatment

Total 10-year cumulative costs of treatment sequences by 1L treatment are summarized in Table 3. Treatment sequences starting with VO had lower 10-year cumulative costs compared to sequences starting with other novel agents (i.e., IBRU, ACAL, acalabrutinib in combination with obinutuzumab [ACAL + O]). For patients without TP53 aberration, the lowest cost sequence starting with VO was VO → VR → VR, provided they had a PFS of at least 12 months after completing the prior therapy. Among patients with TP53 aberration, the lowest cost sequence starting with VO was VO → IBRU → V. 

Among patients without TP53 aberration, cumulative costs per patient of sequences starting with VO ranged from CAD 327,574 to CAD 418,213, whereas sequences starting with IBRU monotherapy or ACAL monotherapy ranged from CAD 772,127 to CAD 930,700. Similarly, in patients with TP53 aberration, cumulative costs per patient of sequences starting with VO ranged from CAD 500,639 to CAD 536,507, while sequences starting with IBRU monotherapy or ACAL monotherapy ranged from CAD 770,737 to CAD 862,554.

Note that patients with TP53 aberration have slightly higher costs for sequences starting with VO compared to patients without TP53, since these patients experience earlier relapses on VO. Therefore, more patients with TP53 aberration require additional lines of treatment, which mostly include continuous BTKis, which are more expensive. This explanation is also applicable for patients with TP53 receiving CLB + O.

#### 3.1.2. First Two Lines of Treatment

**Figure 2:**
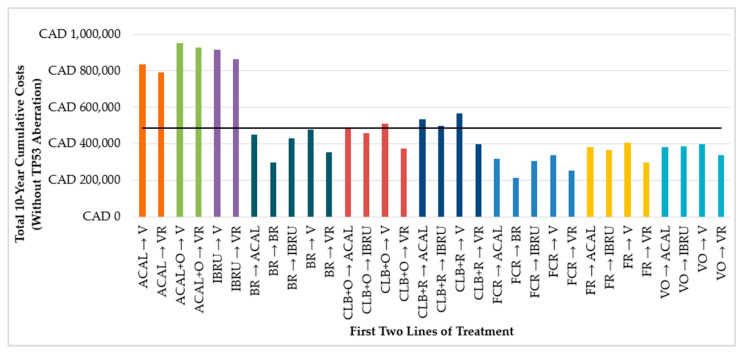
Total 10-year costs of treatment sequences by first two lines of treatment in patients without TP53 aberration. ACAL: acalabrutinib, ACAL + O: acalabrutinib in combination with obinutuzumab, BR: bendamustine in combination with rituximab, CAD: Canadian dollars, CLB + O: chlorambucil in combination with obinutuzumab, CLB + R: chlorambucil in combination with rituximab, FCR: fludarabine, cyclophosphamide, rituximab, FR: fludarabine in combination with rituximab, IBRU: ibrutinib, V: venetoclax, VO: venetoclax in combination with obinutuzumab, VR: venetoclax in combination with rituximab. The black horizontal line represents the mean cost of all treatment sequences.

**Figure 3:**
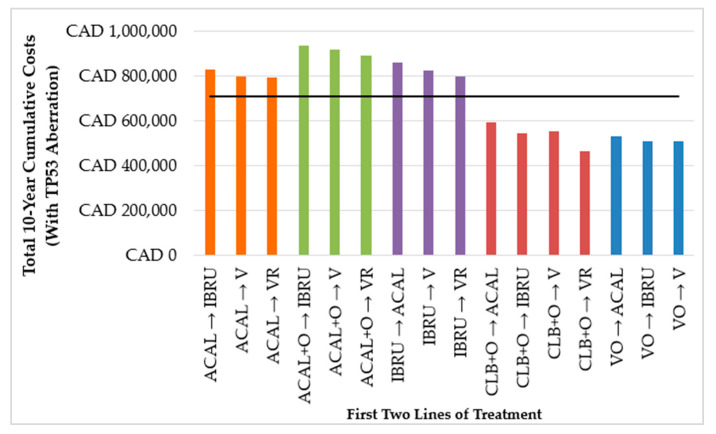
Total 10-year costs of treatment sequences by first two lines of treatment in patients with TP53 aberration. ACAL: acalabrutinib, ACAL + O: acalabrutinib in combination with obinutuzumab, CAD: Canadian dollars, CLB + O: chlorambucil in combination with obinutuzumab, IBRU: ibrutinib, V: venetoclax, VO: venetoclax in combination with obinutuzumab, VR: venetoclax in combination with rituximab. The black horizontal line represents the mean cost of all treatment sequences.

In our model, with a maximum of three lines of treatments over 10 years, cumulative costs for treatment sequences were largely determined by the first two lines of treatment. When comparing treatment sequences starting with the same 1L (e.g., any sequence starting with bendamustine in combination with rituximab [BR]), sequences with continuous BTKis in 2L (e.g., IBRU or ACAL monotherapy) resulted in higher costs compared to sequences with fixed-duration therapies (e.g., VR) in 2L (Figure 2 and Figure 3).

Additionally, among sequences starting with VO, retreatment with a V-based regimen in 2L often resulted in relatively low cumulative costs (from CAD 339,370 to CAD 396,054 for patients without TP53 aberration and CAD 511,641 for patients with TP53 aberration).

#### 3.1.3. VO vs. Non-VO Treatment Sequences

In a scenario assuming that all patients are treated with sequences starting with VO (i.e., VO in 1L for all patients in the model), 10-year cumulative costs were estimated at CAD 359,001 and CAD 518,726 for patients without and with TP53 aberration, respectively. In a scenario assuming that all patients are not treated with sequences with VO (i.e., without VO for all patients), 10-year cumulative costs were estimated at CAD 528,343 and CAD 812,457 for patients without and with TP53 aberration, respectively.

For patients without TP53 aberration, the economic impact of treating all patients with sequences of treatment starting with VO led to 10-year cumulative savings of CAD 169,341 per patient for the Canadian healthcare system. 10-year cumulative savings were higher for patients with TP53 aberration at CAD 293,731 per patient.

## 4. Discussion

The results of this study provide 10-year cumulative cost estimates of various CLL treatment sequences for patients with and without TP53 aberration, in Canada. Overall, sequences starting with VO had lower costs compared to those starting with other novel agents. It was also found that, when comparing treatment sequences starting with the same 1L, sequences with continuous BTKis in 2L incurred greater costs compared to sequences with fixed-duration treatment regimens in 2L. This is an important finding as CLL treatment costs are mostly driven by the first two lines of therapy within the model, considering the inclusion of a maximum of three lines of treatment over a 10-year period. For CLL patients without TP53 aberration and those with TP53 aberration, initiating treatment with VO as 1L was estimated to reduce drug costs by CAD 169,341 and CAD 293,731 per patient, respectively, compared to treatment sequences without VO over 10 years. Given the expected increase in spending on CLL in Canada [8], this study indicates a possible strategy to mitigate these rising costs in a publicly funded health care system. It is also important to note that the projected savings calculated in this study were for the Canadian healthcare system in Canadian dollars; potential savings would be even greater when replicated in a more expensive healthcare system, such as the United States (US).

Prior studies have established the health economic value of VO. In terms of budget impact analysis (BIA), a US study found that the implementation of VO as 1L, including costs associated with AEs, routine care, and monitoring, resulted in cost savings of USD 1.6 million per 1 million members under the US health plan over a 3-year time horizon [59]. Another BIA conducted in France demonstrated that although there is an increase in costs with treatment sequences including VO during the first year, it is followed by cost savings in subsequent years compared to other CLL treatment sequences, resulting in total budget savings of EUR 860 million over 10 years [60]. A US study on CLL treatment sequencing also demonstrated budget savings with VO, with estimated cost reductions of approximately USD 13 million and USD 56 million over 5 and 10 years, respectively, in a hypothetical one-million-member health plan [61]. In terms of cost-effectiveness, a US study by Alrawashdh et al. estimated the 10-year cost-effectiveness of 1L treatments for CLL and determined that CITs yielded less health benefits (i.e., life-years (LYs) and quality-adjusted LYs [QALYs]) at higher costs compared to VO; other targeted therapies were also found to be more costly but resulted in greater health benefits than VO [62]. In another cost-effectiveness study conducted from the Canadian public funding perspective, Chatterjee et al. found that VO is the most cost-effective option in CLL treatment under most willingness-to-pay thresholds. They concluded that VO was a dominant treatment option for unfit 1L CLL patients, meaning that VO accrues higher QALYs and lower total costs, relative to the majority of comparators. Although ACAL accrued higher QALYs than VO, it is also more expensive than VO, and was not found to be cost-effective [63]. 

Note that this study is unique as it is the first to assess the costs of all possible sequences of treatment in CLL, from a Canadian healthcare system perspective. Although the cost-effectiveness study by Chatterjee et al. considered subsequent treatments in their cost data, the cost for each treatment sequence as well as sub-analyses by 1L and 2L treatment were not analyzed. Our study presents data on a granular level, demonstrating the impact of different subsequent treatments on total cumulative costs. Furthermore, this study also assesses the potential savings from treating all Canadian CLL patients with VO in 1L, which has not been previously reported. 

Bruton tyrosine kinase inhibitors have become a treatment of choice for most CLL patients, providing salvage therapy and an alternative treatment option to chemoimmunotherapy. However, now that additional treatment options are available to treat CLL, it is important to assess the impact of treatments on the patients as well as the payers. Consequently, a strength of this study was that it estimated the long-term (i.e., 10-year) budget impact of a number of possible CLL treatment sequences, as opposed to just individual regimens. It also considered a relevant CLL subgroup (i.e., TP53 aberration). This is important as higher-risk patients are expected to incur more costs, which can affect the overall budget impact. Note that at as the time of this analysis, IGHV mutation status was not considered since IGHV testing was not uniform across all Canadian provinces and does not play a major role in the efficacy of novel targeted therapies. The model inputs were retrieved from the most suitable trial data and Canadian resources to ensure that the results would be representative and applicable to the Canadian public health care system. Clinical expert input was also acquired to confirm the validity and accuracy of the model and its input parameters. Additionally, the model included numerous cost items (i.e., treatment acquisition, administrative, monitoring, TLS prophylaxis, AE-related, and support care) to ensure that all costs relevant to manage patients with CLL were considered. 

This study also had some limitations. Allogeneic stem cell transplantation (allo-SCT) is another treatment option for certain patients with CLL [64,65], but it was not included as a possible treatment option in this analysis (only drug therapies were considered). According to key opinion leaders in Canada, allo-SCT is part of the standard of care; however, with access to novel treatments, the number of patients eligible for this treatment is diminishing. Furthermore, although the TP53 aberration subgroup was captured within this study, clinical data were not stratified by TP53 mutation type. It has been reported that patients may have single or multiple TP53 abnormalities, with increased risk for shorter time to therapy as well as increased risk of death with increased number of abnormalities [66,67]. Single clinical data were retrieved and no distinction was made with regards to different TP53 aberration patient profiles, which might not provide accurate costing results for these patients. Another limitation is the exclusion of costs associated with COVID-19 infections. Although not reported in the pivotal clinical trials included in this study, COVID-19 infections represent a significant burden on healthcare costs and clinical admissions related to CLL. Additionally, this model considered that patients treated with oral targeted therapies are seen every three months. However, in clinical practice this frequency varies based on duration of time on therapies and toxicities and will be the subject of future studies as systems adapt to these regimens. Another limitation is that patients could only receive up to three lines of treatment followed by supportive care in this model; however, this may not be reflective of a real-world clinical setting, as patients may also receive further lines of treatment. In addition, the model also disregarded patients who transition to the next line of treatment due to intolerance; only PFS data were used to assess the transition to the following line of treatment, which may underestimate the proportion of patients requiring subsequent lines of treatment. With regards to PFS and OS data, a direct treatment comparison was not available, while an indirect treatment comparison of all treatment comparators would not be feasible and not be justified considering the extent of treatments and lines of treatments included in this analysis. Therefore, a cross-comparison of trials without adjustment was performed, which has its limitations pertaining to the differences within the trials and the populations studied. It was also assumed that drug prices and dosing remained constant over time. Drug prices can change (e.g., generic drug entry) and dose modification may occur throughout a patient’s follow-up, which can, ultimately, impact their treatment costs. Lastly, since data on the proportion of use of treatment sequences are not publicly available, clinical experts provided a hypothetical proportion of use of each treatment sequence, based on funding status across Canada and CLL patient subpopulations. These numbers are hypothetical and may not reflect accurately the entirety of the CLL treatment paradigm in Canada. Health Canada approved the combination of fixed-duration ibrutinib in combination with V in March 2023 based on the GLOW and CAPTIVATE studies [68,69,70]. Although CDA provided a positive recommendation for the reimbursement of this treatment combination in November 2023, it has not yet received public funding in any Canadian jurisdiction [71]. This study does not compare fixed-duration VO to a fixed-duration oral therapy, and it remains to be determined if ibrutinib in combination with V will be cost-effective. Some of these limitations are factors to consider in future research in this area.

## 5. Conclusions

In conclusion, treatment sequences starting with time-limited therapy VO in 1L resulted in lower costs compared to sequences without VO. These savings were mainly due to reductions in the treatment costs associated with a fixed treatment duration.

## Figures and Tables

**Table 1:** Summary of treatment-related parameters.

Treatments	PFS and OS (Transition Probability by 28-Day Cycle) *	Grade 3/4 AEs (%)	Drug Cost ^a^ (CAD/Cycle)	References
**1L**				
FR	Median PFS, 45.0 months (1.4%) Median OS, 105.0 months (0.6%)	Anemia, 4 Neutropenia, 76 Thrombocytopenia, 20 Infection, 20	IV: C1: 3194 C2–6: 3896 Oral: C1: 3620 C2–6: 4322	Woyach, 2011 [38], Byrd, 2003 [41]
FCR	3-year PFS, 72.9% (0.8%) 3-year OS, 91.5% (0.2%)	Anemia, 14.6 Neutropenia, 45 Febrile neutropenia, 15.8 Thrombocytopenia, 15.2 Infection, 9.5 Atrial fibrillation, 1.2	IV: C1: 3071 C2–6: 3773 Oral: C1: 3069 C2–6: 3770	Shanafelt, 2019 [15]
CLB + O	Median PFS, 29.8 months (2.2%) TP53+, median PFS, 11.3 months (5.5%) 5-year OS, 66.0% (0.6%) TP53+, 30-month OS, 85.0% (0.5%)	Anemia, 5 Neutropenia, 35 Thrombocytopenia, 11 Infection, 11	C1: 16,498 C2–6: 5542	Goede, 2018 [26], Moreno, 2019 [27], Goede, 2014 [42]
CLB + R	Median PFS, 15.7 months (4.0%) Median OS, 73.1 months (0.9%)	Anemia, 4 Neutropenia, 27 Thrombocytopenia, 4 Infection, 13	C1: 2169 C2–6: 2871	Goede, 2018 [26], Goede, 2014 [42]
BR	Median PFS, 15.7 months (1.4%) 2-year OS, 95.0% (0.2%)	Anemia, 12 Febrile neutropenia, 7 Infection, 15 Atrial fibrillation, 3	C1: 5720 C2–6: 6421	Woyach, 2018 [43]
IBRU	5-year PFS, 73.0% (0.5%) TP53+, 1-year PFS, 87.0% (1.1%) 5-year OS, 83.0% (0.3%) TP53+, 1-year OS, 89.0% (0.9%)	Anemia, 7 Neutropenia, 13 Infection, 12 Atrial fibrillation, 5	8386	Burger, 2020 [30], Mato, 2018 [32]
VO	5-year PFS, 62.6% (0.7%) TP53+, 5-year PFS, 40.6% (1.4%) 4-year OS, 85.4% (0.3%)	Anemia, 9 Neutropenia, 53 Febrile neutropenia, 5 Thrombocytopenia, 13 Infection, 7 Atrial fibrillation, 2	C1: 16,532 C2: 9153 C3–6: 13,318 C7–13: 7840	Al-Sawaf, 2022 [19], Al-Sawaf, 2020 [44]
ACAL	4-year PFS, 78.0% (0.5%) TP53+, 39-month PFS, 74.0% (0.7%) 4-year OS, 88.0% (0.2%) TP53+, 2-year OS, 95.0% (0.2%)	Neutropenia, 11.2 Bleeding, 2.8 Atrial fibrillation, 1.1	7615	Sharman, 2021 [37], Sharman, 2020 [36]
ACAL + O	4-year PFS, 87.0% (0.3%) TP53+, 33-month PFS, 70.2% (1.0%) 4-year OS, 93.0% (0.1%) TP53+, 2-year OS, 95.0% (0.2%)	Neutropenia, 30.9 Bleeding, 2.9 Atrial fibrillation, 0.6	C1: 7615 C2: 24,048 C3: 13,092 C8+: 7615	Sharman, 2021 [37], Sharman, 2020 [36]
2L/3L **				
FCR	Median PFS, 30.6 months (2.1%) 55-month OS, 60.0% (0.9%)	Anemia, 12 Neutropenia, 42 Febrile neutropenia, 12 Thrombocytopenia, 11 Infection, 5	IV: C1: 3071 C2–6: 3773 Oral: C1: 3069 C2–6: 3770	Robak, 2010 [25]
BR	Median PFS, 21.6 months (2.9%) TP53+, median PFS, 14.6 months (4.3%) 5-year OS, 62.2% (0.7%)	Anemia, 13.8 Neutropenia, 38.8 Febrile neutropenia, 9.6 Thrombocytopenia, 10.1 Infection, 8	C1: 5720 C2–6: 6421	EMA, 2020 [29], Seymour, 2018 [45]
IBRU	Median PFS, 42.5 months (1.5%) TP53+, median PFS, 40.6 months (1.6%) Median OS, 67.7 months (0.9%) TP53+, median OS, 61.8 months (1.0%)	Anemia, 9 Neutropenia, 25 Thrombocytopenia, 10 Bleeding, 10 Infection, 21 Atrial fibrillation, 6	8386	Munir, 2019 [31] Byrd, 2003 [46], Munir, 2019 [31]
V	2-year PFS, 24.0 months (2.3%) TP53+, 1-year PFS, 72.0% (2.5%) 12-month OS, 92.0% (0.6%) TP53+, 2-year OS, 73.0% (1.2%)	Anemia, 29 Neutropenia, 51 Febrile neutropenia, 13 Thrombocytopenia, 29 Infection, 12	C1: 1813 C2+: 7840	EMA, 2020 [29], Jones, 2018 [47]
VR	Median PFS, 55.1 months (1.2%) TP53+, median PFS, 47.9 months (1.3%) 5-year OS, 82.1% (0.3%)	Anemia, 10.8 Neutropenia, 57.7 Febrile neutropenia, 3.6 Thrombocytopenia, 5.7 Infection, 17.5	Ramp-up: 3773 C1: 9945 C2–6: 10,647 C7–26: 7840	EMA, 2020 [29], Seymour, 2018 [45]
ACAL	22-month PFS, 74.7% (1.2%) TP53+, 19-month PFS, 80.2% (1.1%) 12-month OS, 94.0% (0.5%)	Anemia, 11 Neutropenia, 15 Febrile neutropenia, 0 Thrombocytopenia, 4 Infection, 5 Atrial fibrillation, 2	7615	Ghia, 2020 [33]

Descriptions: 1L: first-line, 2L: second-line, 3L: third-line, ACAL: acalabrutinib, ACAL + O: acalabrutinib in combination with obinutuzumab, AEs: adverse events, BR: bendamustine in combination with rituximab, C: cycle, CAD: Canadian dollars, CLB + O: chlorambucil in combination with obinutuzumab, CLB + R: chlorambucil in combination with rituximab, FCR: fludarabine, cyclophosphamide, rituximab, FR: fludarabine in combination with rituximab, IBRU: ibrutinib, OS: overall survival, PFS: progression-free survival, V: venetoclax, VO: venetoclax in combination with obinutuzumab, VR: venetoclax in combination with rituximab. * PFS and OS probabilities extracted from clinical trials were converted to 28-day model cycle probabilities using the conversion equations published by Fleurence (2007) [40]. ** Note that PFS and OS data for 2L and 3L are assumed to be similar. ^a^ all costs are shown in 2022 Canadian dollars.

**Table 2:** Model and cost ^a^ parameters.

Parameters	Model	Reference
**Probabilities, %**		
Patients on IV therapy	100.00	When both formulas are available
**Costs, CAD**		
**Follow-up and laboratory monitoring costs**		
Electrolyte panel	18.08	L226, L204, L053, L165, L194, L061, L700
Renal panel	25.48	L251, L065, L700
Liver function test	21.15	L223, L222, L191, L029, L030, L031, L005, L208, L700
CBC panel	14.74	L393, L700
Coagulation parameters	13.42	L445, L700
Serology	21.01	L319, L700
**Professional fees**		
Consultation, Hematology	168.75	Schedule of Benefits, code A615 [50]
Partial assessment, Hematology	38.05	Schedule of Benefits, code A618 [50]
**Administration costs**		
Physician fee for administration	105.15	Schedule of Benefits, code G359 [50]
Nurse average wage (CAD/min)	0.67 *	Statistic Canada [52]; Job Bank Canada, NOC 3012 [51]
Pharmacist average wage (CAD/min)	0.87 *	Statistic Canada [52]; Job Bank Canada, NOC 3131 [51]
**Adverse events** **^b^**		
Anemia	793.02 *	OCC, code D649 [56] Assuming 2% managed inpatient
Neutropenia	553.32 *	OCC, code D700 [56] Assuming 100% managed outpatient
Febrile neutropenia	10,918.00 *	OCC, code R508 [56] Assuming 100% managed inpatient
Thrombocytopenia	467.27 *	OCC, code D696 [56] Assuming 100% managed outpatient
Bleeding	943.96 *	OCC, code D473 [56] Assuming 4% managed inpatient
Infection	1840.27 *	OCC, code A499/B349 [56] Assuming 25% managed inpatient
Atrial fibrillation	1443.66 *	OCC, code I4890 [56] Assuming 10% managed inpatient
**TLS Prophylaxis**		
VO regimens	1290.44	See Supplementary Material, Tables S7–S9
V and VR regimens	1805.91	See Supplementary Material, Tables S7, S8 and S10
**Palliative care** **^c^**	6103.46/cycle *	De Oliveira (2016) [57]

Descriptions: CAD: Canadian dollars, CBC, complete blood count; IV, intravenous; OCC, Ontario Care Costing; V: venetoclax, VO: venetoclax in combination with obinutuzumab, VR: venetoclax in combination with rituximab. ^a^ All costs are shown in 2022 Canadian dollars. ^b^ Since the numbers of grade 3 and grade 4 AE managed inpatient or outpatient were not available from clinical trials, this proportion was established based on Canadian clinical expert input. ^c^ All patients who transitioned to supportive care were assumed to incur palliative care costs. * Secondary data from Lachaine et al. (2021) were updated for this current study [8].

**Table 3:** Total 10-year costs of treatment sequences by 1L treatment.

1L Treatment		10-Year Cumulative Costs (2022 CAD)	
		Without TP53 Aberration	With TP53 Aberration
BTKis	ACAL	772,127–854,077	770,737–832,182
	ACAL + O	914,811–966,247	890,042–942,126
	IBRU	844,607–930,700	789,732–862,554
Chemoimmunotherapy	BR	264,460–486,313	*
	CLB + O	346,879–519,606	448,574–603,645
	CLB + R	365,926–577,797	*
	FCR	190,282–343,317	*
	FR	277,979–413,126	*
BCL-2i	VO	327,574–418,213	500,639–536,507

Descriptions: 1L: first-line, ACAL: acalabrutinib, ACAL + O: acalabrutinib in combination with obinutuzumab, BCL-2i: B-cell lymphoma 2 inhibitor, BR: bendamustine in combination with rituximab, BTKis: Bruton tyrosine kinase inhibitors, CAD: Canadian dollars, CLB + O: chlorambucil in combination with obinutuzumab, CLB + R: chlorambucil in combination with rituximab, FCR: fludarabine, cyclophosphamide, rituximab, FR: fludarabine in combination with rituximab, IBRU: ibrutinib, VO: venetoclax in combination with obinutuzumab. * Not commonly used in patients with TP53 aberration, therefore not considered as a potential 1L treatment in the study.

## Data Availability

The original contributions presented in the study are included in the article/Supplementary Material, further inquiries can be directed to the corresponding author/s.

## Supplementary Materials

The following supporting information can be downloaded at: https://www.mdpi.com/article/10.3390/cancers16183182/s1, Table S1. Treatment Sequence Utilization for Patients without TP53 Aberration; Table S2. Treatment Sequence Utilization for Patients with TP53 Aberration; Table S3. Treatment Acquisition Costs; Table S4. Administration Costs per Treatment; Table S5. Professional Fee Cost per Treatment; Table S6. Laboratory Monitoring Frequencies and Cost per Treatment; Table S7. TLS Risk Distribution; Table S8. Resources used for TLS Prophylaxis Unit Cost; Table S9. TLS Prophylaxis Algorithm Applied to VO Regimen; Table S10. TLS Prophylaxis Algorithm Applied to V/VR Regimens; Table S11. Severe AE Frequencies per Treatment—1L; Table S12. Severe AE Frequencies per Treatment—2L/3L
