# Supplementary material for: The Economic Impact of Treatment Sequencing in Chronic Lymphocytic Leukemia in Canada Using Venetoclax plus Obinutuzumab
Source: Cancers (Basel). 2024 Sep 17;16(18):3182. doi: 10.3390/cancers16183182 (PMC11430142; doi:10.3390/cancers16183182)
Supplement: Supplementary file 1 [file cancers-16-03182-s001.zip › cancers-3170884-supplementary.pdf]

Supplementary Materials

Table S1. Treatment Sequence Utilization for Patients without TP53 Aberration

No. Treatment Sequence	Treatment Sequence	Proportion of use (%)	
		Including VO in 1L	Excluding VO in 1L
**1**	ACAL - V - BR	3.5%	5.6%
**2**	ACAL - V - IBRU	0.0%	0.0%
**3**	ACAL - VR - BR	0.0%	0.0%
**4**	ACAL - VR - IBRU	0.7%	1.1%
**5**	ACAL - VR – V	8.8%	14.0%
**6**	ACAL+O - V - BR	0.0%	0.0%
**7**	ACAL+O - V - IBRU	0.0%	0.0%
**8**	ACAL+O - VR - BR	0.0%	0.0%
**9**	ACAL+O - VR - IBRU	0.0%	0.0%
**10**	ACAL+O - VR - V	0.0%	0.0%
**11**	BR - ACAL - V	1.4%	1.4%
**12**	BR - ACAL - VR	0.0%	0.0%
**13**	BR - BR - ACAL	0.0%	0.0%
**14**	BR - BR - IBRU	0.0%	0.0%
**15**	BR - BR - V	0.0%	0.0%
**16**	BR - BR - VR	0.5%	0.5%
**17**	BR - IBRU - V	1.4%	1.4%
**18**	BR - IBRU - VR	0.0%	0.0%
**19**	BR - V - ACAL	0.5%	0.5%
**20**	BR - V - IBRU	0.5%	0.5%
**21**	BR - VR - ACAL	0.9%	0.9%
**22**	BR - VR - IBRU	0.9%	0.9%
**23**	BR - VR - V	0.5%	0.5%
**24**	BR - VR - VR	0.9%	0.9%
**25**	CLB+O - ACAL - V	3.6%	7.9%
**26**	CLB+O - ACAL - VR	1.3%	2.8%
**27**	CLB+O - IBRU - V	1.0%	2.3%
**28**	CLB+O - IBRU - VR	1.3%	2.8%
**29**	CLB+O - V - ACAL	1.5%	3.4%
**30**	CLB+O - V - IBRU	0.3%	0.6%
**31**	CLB+O - VR - ACAL	2.3%	5.1%
**32**	CLB+O - VR - IBRU	2.3%	5.1%
**33**	CLB+O - VR - V	0.3%	0.6%
**34**	CLB+O - VR - VR	0.3%	0.6%
**35**	CLB+R - ACAL - V	0.0%	0.0%
**36**	CLB+R - ACAL - VR	0.0%	0.0%
**37**	CLB+R - IBRU - V	0.0%	0.0%
**38**	CLB+R - IBRU - VR	0.0%	0.0%
**39**	CLB+R - V - ACAL	0.5%	0.5%
**40**	CLB+R - V - IBRU	0.0%	0.0%
**41**	CLB+R – VR - ACAL	2.3%	2.3%
**42**	CLB+R - VR - IBRU	0.0%	0.0%

**43**	CLB+R - VR - V	0.0%	0.0%
**44**	CLB+R - VR - VR	0.0%	0.0%
**45**	FCR - ACAL - V	1.4%	1.4%
**46**	FCR - ACAL - VR	2.3%	2.3%
**47**	FCR - BR - ACAL	1.4%	1.4%
**48**	FCR - BR - IBRU	0.0%	0.0%
**49**	FCR - BR - V	0.5%	0.5%
**50**	FCR - BR - VR	0.0%	0.0%
**51**	FCR - IBRU - V	1.4%	1.4%
**52**	FCR - IBRU - VR	2.3%	2.3%
**53**	FCR - V - ACAL	2.4%	2.4%
**54**	FCR - V - IBRU	0.0%	0.0%
**55**	FCR - VR - ACAL	5.2%	5.2%
**56**	FCR - VR - IBRU	2.9%	2.9%
**57**	FCR - VR - V	0.5%	0.5%
**58**	FCR - VR - VR	0.5%	0.5%
**59**	FR - ACAL - V	3.7%	3.7%
**60**	FR - ACAL - VR	0.0%	0.0%
**61**	FR - IBRU - V	2.3%	2.3%
**62**	FR - IBRU - VR	0.0%	0.0%
**63**	FR - V - ACAL	0.0%	0.0%
**64**	FR - V - IBRU	0.0%	0.0%
**65**	FR - VR - ACAL	0.0%	0.0%
**66**	FR - VR - IBRU	0.0%	0.0%
**67**	FR - VR - V	0.0%	0.0%
**68**	FR - VR - VR	0.0%	0.0%
**69**	IBRU - V - ACAL	0.0%	0.0%
**70**	IBRU - V - BR	1.8%	3.5%
**71**	IBRU - V - IBRU	0.4%	0.7%
**72**	IBRU - VR - BR	1.8%	3.5%
**73**	IBRU - VR - V	1.8%	3.5%
**74**	VO - ACAL - BR	0.2%	0.0%
**75**	VO - ACAL - V	2.3%	0.0%
**76**	VO - ACAL - VR	0.0%	0.0%
**77**	VO - IBRU - BR	0.2%	0.0%
**78**	VO - IBRU - V	0.2%	0.0%
**79**	VO - IBRU - VR	0.2%	0.0%
**80**	VO - V - ACAL	0.2%	0.0%
**81**	VO - V - BR	0.2%	0.0%
**82**	VO - V - IBRU	4.7%	0.0%
**83**	VO - VR - ACAL	10.5%	0.0%
**84**	VO - VR - IBRU	10.5%	0.0%
**85**	VO - VR - V	0.2%	0.0%
**86**	VO - VR - VR	0.2%	0.0%

ACAL: acalabrutinib, ACAL+O: acalabrutinib in combination with obinutuzumab, BR: bendamustine in combination with rituximab, CLB+O: chlorambucil in combination with obinutuzumab, CLB+R: chlorambucil in combination with rituximab, FCR: fludarabine, cyclophosphamide, rituximab, FR: fludarabine in combination with rituximab, IBRU: ibrutinib V: venetoclax, VO: venetoclax in combination with obinutuzumab, VR: venetoclax in combination with rituximab.

Table S2. Treatment Sequence Utilization for Patients with TP53 Aberration

No. Treatment Sequence	Treatment Sequence	Proportion of use (%)	
		Including VO in 1L	Excluding VO in 1L
**1**	ACAL - IBRU - V	0.7%	0.7%
**2**	ACAL - IBRU - VR	0.7%	0.7%
**3**	ACAL - V - BR	9.0%	10.1%
**4**	ACAL - V - FCR	9.0%	10.1%
**5**	ACAL - V - IBRU	9.0%	10.1%
**6**	ACAL - VR - IBRU	3.4%	3.8%
**7**	ACAL - VR - V	3.4%	3.8%
**8**	ACAL+O - IBRU - V	0.0%	0.0%
**9**	ACAL+O - IBRU - VR	0.0%	0.0%
**10**	ACAL+O - V - BR	2.4%	11.6%
**11**	ACAL+O - V - IBRU	2.4%	11.6%
**12**	ACAL+O - VR - IBRU	0.1%	0.6%
**13**	ACAL+O - VR - V	0.1%	0.6%
**14**	CLB+O - ACAL - IBRU	0.5%	0.7%
**15**	CLB+O - ACAL - V	0.5%	0.7%
**16**	CLB+O - ACAL - VR	0.5%	0.7%
**17**	CLB+O - IBRU - ACAL	0.5%	0.7%
**18**	CLB+O - IBRU - V	0.5%	0.7%
**19**	CLB+O - IBRU - VR	0.5%	0.7%
**20**	CLB+O - V - ACAL	0.5%	0.7%
**21**	CLB+O - V - IBRU	0.5%	0.7%
**22**	CLB+O - VR - ACAL	0.5%	0.7%
**23**	CLB+O - VR - IBRU	0.5%	0.7%
**24**	CLB+O - VR - V	0.5%	0.7%
**25**	IBRU - ACAL - V	0.9%	0.7%
**26**	IBRU - ACAL - VR	0.9%	0.7%
**27**	IBRU - V - ACAL	13.3%	10.7%
**28**	IBRU - V - BR	13.3%	10.7%
**29**	IBRU - VR - ACAL	3.3%	2.7%
**30**	IBRU - VR - V	3.3%	2.7%
**31**	VO - ACAL - IBRU	3.3%	0.0%
**32**	VO - ACAL - V	3.3%	0.0%
**33**	VO - IBRU - ACAL	3.3%	0.0%
**34**	VO - IBRU - V	3.3%	0.0%
**35**	VO - V - ACAL	3.3%	0.0%
**36**	VO - V - IBRU	3.3%	0.0%

ACAL: acalabrutinib, ACAL+O: acalabrutinib in combination with obinutuzumab, BR: bendamustine in combination with rituximab, CLB+O: chlorambucil in combination with obinutuzumab, FCR: fludarabine, cyclophosphamide, rituximab, IBRU: ibrutinib V: venetoclax, VO: venetoclax in combination with obinutuzumab, VR: venetoclax in combination with rituximab.

Table S3. Treatment Acquisition Costs

Product	Administration	Strength	Recommended Dose	Cost per mg	Cost of Regimen per Cycle
**CLB+O**					
O	IV	25 mg/ml (40 ml)	C1 1000 mg on D1, D8 & D15; C2 to C6 1000mg on D1	\$5.48	C1: \$16,497.97<br>C2-6: \$5,542.29
CLB	Oral	2 mg	0.5 mg/kg D1 & D15 of each cycle, C1 to C6	\$0.85	
**CLB+R**					
R	IV	100 mg<br>500 mg	C1 375 mg/m^2; C2 to C6 500 mg/m2	\$2.97<br>\$2.97	C1: \$2,169.44<br>C2-6: \$2,871.10
CLB	Oral	2 mg	0.5 mg/kg D1 & D15 of each cycle, C1 to C6	\$0.85	
**FCR (IV)**					
F	IV	25 mg/ml (2ml)	25 mg/m^2 once per day, days 1 to 3, from C1 to C6	\$4.61	C1: \$3,070.86<br>C2-6: \$3,772.52
C	IV	200 mg<br>500 mg<br>1000 mg<br>2000 mg	250 mg/m^2/day, days 1 to 3, from C1 to C6	\$0.37<br>\$0.19<br>\$0.17<br>\$0.16	
R	IV	100 mg<br>500 mg	C1 375 mg/m^2; C2 to C6 500 mg/m2	\$2.97<br>\$2.97	
**FCR (Oral)**					
F	Oral	10 mg	25 mg/m^2 once daily, days 1 to 5, from C1 to C6	\$4.01	C1: \$3,068.53<br>C2-6: \$3,770.19
C	Oral	25 mg<br>50 mg	150 mg/m^2/day, days 1 to 5, from C1 to C6	\$0.01<br>\$0.01	
R	IV	100 mg<br>500 mg	C1 375 mg/m^2; C2 to C6 500 mg/m^2	\$2.97<br>\$2.97	
**FR (IV)**					
F	IV	25 mg/ml (2ml)	IV: 25 mg/m^2, days 1 to 5, C1 to C6	\$4.61	C1: \$3,194.10<br>C2-6: \$3,895.76
R	IV	100 mg<br>500 mg	C1 375 mg/m^2; C2 to C6 500 mg/m^2	\$2.97<br>\$2.97	
**FR (Oral)**					
F	Oral	10 mg	Oral: 40 mg/m^2 once daily, days 1 to 5, C1 to C6	\$4.01	C1: \$3,619.86<br>C2-6: \$4,321.52
R	IV	100 mg<br>500 mg	C1 375 mg/m^2; C2 to C6 500 mg/m^2	\$2.97<br>\$2.97	
**BR**					
B	IV	25 mg<br>100 mg	90 mg/m^2 D1 & D2, C1 to C6	\$10.63<br>\$10.63	C1: \$5,719.65<br>C2-6: \$6,421.31
R	IV	100 mg<br>500 mg	C1 375 mg/m^2; C2 to C6 500 mg/m^2	\$2.97<br>\$2.97	
**IBRU**					
IBRU	Oral	140 mg	420 mg daily	\$0.71	\$8,386.14
**V**					
V	Oral	Ramp up kit<br>10 mg<br>50 mg<br>100 mg	Week 1 20 mg/day, week 2 50 mg/day, week 3 100 mg/day, week 4 200 mg/day, week 5 and beyond 400 mg/day	\$1,813 (\$/kit)<br>\$0.70<br>\$0.70<br>\$0.70	C1: \$1,813.00<br>C2+: \$7,840.00
**VR**					
V	Oral	Ramp up kit<br>10 mg<br>50 mg<br>100 mg	Week 1 20 mg/day, week 2 50 mg/day, week 3 100 mg/day, week 4 200 mg/day, week 5 and beyond 400 mg/day, for 2 years	\$1,813 (\$/kit)<br>\$0.70<br>\$0.70	Ramp-up: \$3,773.00<br>C1: \$9,944.99<br>C2-6: \$10,646.65

Product	Administration	Strength	Recommended Dose	Cost per mg	Cost of Regimen per Cycle
				$0.70	C7-26: $7,840.00
R	IV	100 mg<br>500 mg	C1 375 mg/m^2 D1, C2 to C6 500 mg/m^2 D1	$2.97<br>$2.97	
**VO**					
V	Oral	Ramp up kit<br>10 mg<br>50 mg<br>100 mg	Dose ramp-up period (5 weeks total) starting day 22 of cycle 1 (week 1 20 mg/day, week 2 50 mg/day, week 3 100 mg/day, week 4 200 mg/day, week 5 400 mg/day). Afterwards, keep 400 mg/day, for 1 year	$1,813 ($/kit)<br>$0.70<br>$0.70<br>$0.70	C1: $16,531.52<br>C2: $9,152.84<br>C3-6: $13,317.84<br>C7-13: $7,840.00
O	IV	25 mg/ml (40 ml)	C1 1000 mg on D1, D8 & D15; C2 to C6 1000 mg on D1	$5.48	
**ACAL**					
ACAL	Oral	100 mg	100 mg twice daily, until disease progression	$1.36	$7,614.60
**ACAL+O**					
ACAL	Oral	100 mg	100 mg twice daily, until disease progression	$1.36	C1: $7,614.60<br>C2: $24,048.12<br>C3: $13,092.44<br>C8+: $7,614.60
O	IV	25 mg/ml (40 ml)	C2 1000 mg on D1, D8 & D15; C3 to C7 1000 mg on D1	$5.48	

ACAL: Acalabrutinib, ACAL+O: acalabrutinib in combination with obinutuzumab, BR: bendamustine in combination with rituximab, C: cycle, CLB+O: chlorambucil in combination with obinutuzumab, CLB+R: chlorambucil in combination with rituximab, D: day, F: fludarabine, FCR: fludarabine, cyclophosphamide, rituximab, FR: fludarabine in combination with rituximab, IBRU: ibrutinib, IV: intravenous, V: venetoclax, VO: venetoclax in combination with obinutuzumab, VR: venetoclax in combination with rituximab.

Table S4. Administration Costs per Treatment

Administration Costs per Treatment		Frequency per Cycle		
**FC (IV) R**	**Minutes**	**C1**	**C2 to C6**	**Maintenance**
Physician cost	-	3	3	0
Pharmacy workload (Average per visit; min)	26.889	3	3	0
Nursing workload (Average per visit; min)	54.389	3	3	0
**Average cost per 28-day cycle**		**$494.50**	**$494.50**	**$0.00**
**FC (PO) R**	**Minutes**	**C1**	**C2 to C6**	**Maintenance**
Physician cost	-	1	1	0
Pharmacy workload (Average per visit; min)	28.643	1	1	0
Nursing workload (Average per visit; min)	84.167	1	1	0
**Average cost per 28-day cycle**		**$186.21**	**$186.21**	**$0.00**
**F (IV) R**	**Minutes**	**C1**	**C2 to C6**	**Maintenance**
Physician cost	-	5	5	0
Pharmacy workload (Average per visit; min)	10.148	5	5	0
Nursing workload (Average per visit; min)	44.167	5	5	0
**Average cost per 28-day cycle**		**$717.18**	**$717.18**	**$0.00**
**F (PO) R**	**Minutes**	**C1**	**C2 to C6**	**Maintenance**
Physician cost	-	1	1	0
Pharmacy workload (Average per visit; min)	20.946	1	1	0
Nursing workload (Average per visit; min)	69.167	1	1	0
**Average cost per 28-day cycle**		**$169.51**	**$169.51**	**$0.00**
**F (IV)**	**Minutes**	**C1**	**C2 to C6**	**Maintenance**
Physician cost	-	5	5	**0**
Pharmacy workload (Average per visit; min)	8.059	5	5	0
Nursing workload (Average per visit; min)	36.667	5	5	0
**Average cost per 28-day cycle**		**$683.08**	**$683.08**	**$0.00**
**F (PO)**		**C1**	**C2 to C6**	**Maintenance**
Physician cost	-	0	0	0
**Average cost per 28-day cycle**		**$0.00**	**$0.00**	**$0.00**
**CLB+R**	**Minutes**	**C1**	**C2 to C6**	**Maintenance**
Physician cost	-	1	1	0
Pharmacy workload (Average per visit; min)	20.946	1	1	0
Nursing workload (Average per visit; min)	69.167	1	1	0
**Average cost per 28-day cycle**		**$169.51**	**$169.51**	**$0.00**
**CLB+O**	**Minutes**	**C1**	**C2 to C6**	**Maintenance**
Physician cost	-	3	1	0
Pharmacy workload (Average per visit; min)	18.249	3	1	0
Nursing workload (Average per visit; min)	74.833	3	1	0
**Average cost per 28-day cycle**		**$512.81**	**$170.94**	**$0.00**
**BR**	**Minutes**	**C1**	**C2 to C6**	**Maintenance**
Physician cost	-	2	2	0
Pharmacy workload (Average per visit; min)	24.073	2	2	0
Nursing workload (Average per visit; min)	55.417	2	2	0
**Average cost per 28-day cycle**		**$326.13**	**$326.13**	**$0.00**
**ACAL+O**	**Minutes**	**C1**	**C2**	**C3 to C7**
Physician cost	-	0	3	1
Pharmacy workload (Average per visit; min)	18.249	0	3	1

Administration Costs per Treatment		Frequency per Cycle		
Nursing workload (Average per visit; min)	74.833	0	3	1
**Average cost per 28-day cycle**		**$0.00**	**$512.81**	**$170.94**
**VR**	**Minutes**	**C1**	**C2 to C6**	**Maintenance**
Physician cost	-	1	1	0
Pharmacy workload (Average per visit; min)	20.946	1	1	0
Nursing workload (Average per visit; min)	69.167	1	1	0
**Average cost per 28-day cycle**		**$169.51**	**$169.51**	**$0.00**
**VO**	**Minutes**	**C1**	**C2 to C6**	**Maintenance**
Physician cost	-	3	1	0
Pharmacy workload (Average per visit; min)	18.249	3	1	0
Nursing workload (Average per visit; min)	74.833	3	1	0
**Average cost per 28-day cycle**		**$512.81**	**$170.94**	**$0.00**

ACAL+O: acalabrutinib in combination with obinutuzumab, BR: bendamustine in combination with rituximab, C: cycle CLB+O: chlorambucil in combination with obinutuzumab, CLB+R: chlorambucil in combination with rituximab, F: fludarabine, FCR: fludarabine, cyclophosphamide, rituximab, FR: fludarabine in combination with rituximab, IV: intravenous, PO: Per os, VO: venetoclax in combination with obinutuzumab, VR: venetoclax in combination with rituximab.

Table S5. Professional Fee Cost per Treatment

Professional Fee Cost per Treatment	C1*	C2 to C6	Maintenance
**Non-V-based treatment**			
Visit, Hematology	2	1	Once every 6 months
**Cost per 28 days**	**$206.80**	**$38.05**	**$6.34**
**V-based Treatment**			
Visit, Hematology	4	1	Once every 6 months
**Cost per 28 days**	**$282.90**	**$38.05**	**$6.34**

C: cycle, V: venetoclax.

*Only one consultation can be charged per patient. All subsequent visits are charged as partial assessments.

Table S6. Laboratory Monitoring Frequencies and Cost per Treatment

Treatment/Healthcare Service	Frequency per Cycle			
**FCR / FR**	**C1**	**C2 to C6**	**Maintenance**	
CBC Panel	2	1	Once every 6 months	
Liver function tests	2	1	Once every 6 months	
Renal panel	2	1	Once every 6 months	
Serology	1	0	0	
**Total lab results per 28-day cycle**	**$143.75**	**$61.37**	**$10.23**	
**F**	**C1**	**C2 to C6**	**Maintenance**	
CBC Panel	1	1	Once every 6 months	
Liver function tests	1	1	Once every 6 months	
**Total lab results per 28-day cycle**	**$35.89**	**$35.89**	**$5.98**	
**CLB+R / CLB+O**	**C1**	**C2 to C6**	**Maintenance**	
CBC Panel	2	2	Once every 6 months	
Liver function tests	1	1	Once every 6 months	
Renal panel	1	1	Once every 6 months	
Serology	1	0	0	
**Total lab results per 28-day cycle**	**$97.12**	**$61.37**	**$10.23**	
**BR**	**C1**	**C2 to C6**	**Maintenance**	
CBC Panel	1	1	Once every 6 months	
Electrolyte	1	1	Once every 6 months	
Liver function tests	1	1	Once every 6 months	
Renal panel	1	1	Once every 6 months	
Serology	1	0	0	
**Total lab results per 28-day cycle**	**$100.46**	**$79.45**	**$13.24**	
**IBRU**	**C1**	**C2 to C6**	**Maintenance**	
CBC Panel	4	1	Once every 3 months	
Liver function tests	4	1	Once every 3 months	
Renal panel	4	1	Once every 3 months	
INR	4	0	0	
**Total lab results per 28-day cycle**	**$299.16**	**$61.37**	**$20.46**	
**ACAL / ACAL+O**	**C1**	**C2 to C6**	**Maintenance**	
CBC Panel	2	1	Once every 3 months	
Liver function tests	2	1	Once every 3 months	
Renal panel	2	1	Once every 3 months	
**Total lab results per 28-day cycle**	**$97.26**	**$62.01**	**$20.46**	
**V / VR**	**C1**	**C2**	**C3 to C6**	**Maintenance**
CBC Panel	4	1	1	Once every 3 months
Liver function tests	8	2	1	Once every 3 months
Renal panel	8	2	1	Once every 3 months
Electrolyte panel	8	2	1	Once every 3 months
INR	1	1	1	0
**Total lab results per 28-day cycle**	**$590.06**	**$157.58**	**$92.87**	**$26.48**
**VO**	**C1**	**C2**	**C3 to C6**	**Maintenance**

Treatment/Healthcare Service	Frequency per Cycle			
CBC Panel	2	3	1	Once every 3 months
Liver function tests	3	5	1	Once every 3 months
Renal panel	3	5	1	Once every 3 months
Electrolyte panel	3	5	1	Once every 3 months
INR	1	1	1	0
**Total lab results per 28-day cycle**	**$237.03**	**$381.19**	**$92.87**	**$26.48**

ACAL: acalabrutinib, ACAL+O: acalabrutinib in combination with obinutuzumab, BR: bendamustine in combination with rituximab, C: Cycle, CBC: complete blood count, CLB+O : chlorambucil in combination with obinutuzumab CLB+R: chlorambucil in combination with rituximab, F: fludarabine, FCR: fludarabine, cyclophosphamide, rituximab, FR: fludarabine in combination with rituximab, IBRU: ibrutinib, INR: international normalized ratio, V: venetoclax, VO: venetoclax in combination with obinutuzumab, VR: venetoclax in combination with rituximab.

Table S7. TLS Risk Distribution

Treatment	Low/Moderate Risk	High Risk	Source
VO	78.0%	22.0%	Al-Sawaf, 2020 [1]
V	58.0%	42.0%	Stilgenbauer, 2016 [2]
VR	72.1%	27.9%	Seymour, 2018 [3]

TLS: tumour lysis syndrome, V: venetoclax, VO: venetoclax in combination with obinutuzumab, VR: venetoclax in combination with rituximab.

Table S8. Resources used for TLS Prophylaxis Unit Cost

Resource	Unit Cost	Source
Renal panel	$25.48	Ontario Schedule of Benefits for Laboratory Services, code L700, L251, L065.[4]
Uric acid test	$11.92	Ontario Schedule of Benefits for Laboratory Services, code L700, L252.[4]
Allopurinol (mg)	$0.0007	ODB Formulary, DIN 02396343, 300 mg tablet.[4]
Rasburicase (mg)	$95.67	IQVIA Pharmastat
Rasburicase infusion cost	$143.86	Assuming 10 min preparation (pharmacist cost – Job bank Canada NOC 3131), 30 min infusion and 15 min observation (nurse cost – job bank Canada NOC 3012), as well as a physician supervision fee.[5]
IV hydration with 3L 5% dextrose normal saline, Cost per day	$10.00	Assumption
Cost per CT scan	$86.60	Ontario Schedule of Benefits for Physician Services, code X125.[6]
Cost per inpatient day	$1,292.50	CIHI, Patient cost estimator (code 626). Inflated to $2022 using CPI.[7]
Cost per physician inpatient visit	$38.05	Ontario Schedule of Benefits for Physician Services, code A618.[6]
Cost per physician outpatient visit	$38.05	Ontario Schedule of Benefits for Physician Services, code A618.[6]
Cost per nurse visit	$40.00	Assuming 1 hour per visit. Average nursing wage retrieved from Job Bank Canada, NOC 3012.[5]

CIHI: Canadian institute for health information, CPI: consumer price index, CT: computed tomography, IV: intravenous, ODB: Ontario Drug Benefit, TLS: tumour lysis syndrome.

Table S9. TLS Prophylaxis Algorithm Applied to VO Regimen

Week	1	2	3	4	5	Total	Cost ($)	Weighted Average Cost
**Lower risk**								
Renal panel	3.00	3.00	3.00	3.00	3.00	15.00	$382.20	
Uric acid test	3.00	3.00	3.00	3.00	3.00	15.00	$178.80	
Allopurinol (mg)	3,000.00	2,100.00	2,100.00	2,100.00	2,100.00	1,1400.00	$8.08	
Rasburicase (mg)	0.00	0.00	0.00	0.00	0.00	0.00	$0.00	
Rasburicase infusion cost	0.00	0.00	0.00	0.00	0.00	0.00	$0.00	
IV hydration with saline, Cost per day	0.00	0.00	0.00	0.00	0.00	0.00	$0.00	
Cost per CT scan	1.00	0.00	0.00	0.00	0.00	1.00	$86.60	
Cost per inpatient day	0.00	0.00	0.00	0.00	0.00	0.00	$0.00	
Cost per physician inpatient visit	0.00	0.00	0.00	0.00	0.00	0.00	$0.00	
Cost per physician outpatient visit	1.00	1.00	1.00	1.00	1.00	5.00	$190.25	
Cost per nurse visit	1.00	1.00	1.00	1.00	1.00	5.00	$200.00	
**Greater risk**								**$1,290.44**
Renal panel	3.26	3.26	3.26	3.26	3.26	16.30	$415.32	
Uric acid test	3.26	3.26	3.26	3.26	3.26	16.30	$194.30	
Allopurinol (mg)	3,000.00	2,100.00	2,100.00	2,100.00	2,100.00	1,1400.00	$8.08	
Rasburicase (mg)	3.65	0.00	0.00	0.00	0.00	3.65	$349.20	
Rasburicase infusion cost	0.24	0.00	0.00	0.00	0.00	0.24	$34.53	
IV hydration with saline, Cost per day	1.00	1.00	0.26	0.26	0.26	2.78	$27.80	
Cost per CT scan	1.00	0.00	0.00	0.00	0.00	1.00	$86.60	
Cost per inpatient day	0.26	0.26	0.00	0.00	0.00	0.52	$672.10	
Cost per physician inpatient visit	0.26	0.26	0.00	0.00	0.00	0.52	$19.79	
Cost per physician outpatient visit	0.74	0.74	1.00	1.00	1.00	4.48	$170.46	
Cost per nurse visit	0.74	0.74	1.00	1.00	1.00	4.48	$179.20	

CT: computed tomography, IV: intravenous; TLS: tumour lysis syndrome, VO: venetoclax in combination with obinutuzumab.

Table S10. TLS Prophylaxis Algorithm Applied to V/VR Regimens

Week	1	2	3	4	5	Total	Cost ($)	Weighted Average Cost
**Lower risk**								
Renal panel	3.00	3.00	3.00	3.00	3.00	15.00	$382.20	
Uric acid test	3.00	3.00	3.00	3.00	3.00	15.00	$178.80	
Allopurinol (mg)	6,000.00	4,200.00	4,200.00	4,200.00	4,200.00	22,800.00	$16.15	
Rasburicase (mg)	0.00	0.00	0.00	0.00	0.00	0.00	$0.00	
Rasburicase infusion cost	0.00	0.00	0.00	0.00	0.00	0.00	$0.00	
IV hydration with saline, Cost per day	0.00	0.00	0.00	0.00	0.00	0.00	$0.00	
Cost per CT scan	1.00	0.00	0.00	0.00	0.00	1.00	$86.60	
Cost per inpatient day	0.00	0.00	0.00	0.00	0.00	0.00	$0.00	
Cost per physician inpatient visit	0.00	0.00	0.00	0.00	0.00	0.00	$0.00	
Cost per physician outpatient visit	1.00	1.00	1.00	1.00	1.00	5.00	$190.25	
Cost per nurse visit	1.00	1.00	1.00	1.00	1.00	5.00	$200.00	
**Greater risk**								**$1,805.91**
Renal panel	3.26	3.26	3.26	3.26	3.26	16.30	$415.32	
Uric acid test	3.26	3.26	3.26	3.26	3.26	16.30	$194.30	
Allopurinol (mg)	6,000.00	4,200.00	4,200.00	4,200.00	4,200.00	22,800.00	$16.15	
Rasburicase (mg)	0.98	0.00	0.00	0.00	0.00	0.98	$93.76	
Rasburicase infusion cost	0.06	0.00	0.00	0.00	0.00	0.06	$8.63	
IV hydration with saline, Cost per day	0.78	0.78	0.78	0.78	0.78	3.90	$39.00	
Cost per CT scan	1.00	0.00	0.00	0.00	0.00	1.00	$86.60	
Cost per inpatient day	1.00	1.00	0.00	0.00	0.00	2.00	$2,585.00	
Cost per physician inpatient visit	1.00	1.00	0.00	0.00	0.00	2.00	$76.10	
Cost per physician outpatient visit	0.00	0.00	1.00	1.00	1.00	3.00	$114.15	
Cost per nurse visit	0.00	0.00	1.00	1.00	1.00	3.00	$120.00	

CT: computed tomography, **IV:** intravenous, TLS: tumour lysis syndrome, V: venetoclax, VR: venetoclax in combination with rituximab.

Table S11. Severe AE Frequencies per Treatment – 1L

Treatment	Adverse Events	%	Weighted Average Cost	Total Cost per Patient	Source
**F**	Anemia	15.0%	$118.95	$1,728	Eichhorst, 2009 [8]
	Neutropenia	12.0%	$66.40		
	Febrile neutropenia	0.0%	$0.00		
	Thrombocytopenia	15.0%	$70.09		
	Bleeding	0.0%	$0.00		
	Infection	80.0%	$1,472		
	Arterial fibrillation	0.0%	$0.00		
**FR**	Anemia	4.0%	$31.72	$913.75	Byrd, 2003 [9]
	Neutropenia	76.0%	$420.52		
	Febrile neutropenia	0.0%	$0.00		
	Thrombocytopenia	20.0%	$93.45		
	Bleeding	0.0%	$0.00		
	Infection	20.0%	$368.05		
	Arterial fibrillation	0.0%	$0.00		
	Neutropenia	45.0%	$248.99		
	Febrile neutropenia	15.8%	$1,725.04		
	Thrombocytopenia	15.2%	$71.02		
	Bleeding	0.0%	$0.00		
	Infection	9.5%	$174.83		
	Arterial fibrillation	1.2%	$17.32		
**CLB+O**	Anaemia	5.0%	$39.65	$487.14	Goede, 2014 [10]
	Neutropenia	35.0%	$193.66		
	Febrile neutropenia	0.0%	$0.00		
	Thrombocytopenia	11.0%	$51.40		
	Bleeding	0.0%	$0.00		
	Infection	11.0%	$202.43		
	Arterial fibrillation	0.0%	$0.00		
**CLB+R**	Anaemia	4.0%	$31.72	$439.04	Goede, 2014 [10]
	Neutropenia	27.0%	$149.40		
	Febrile neutropenia	0.0%	$0.00		
	Thrombocytopenia	4.0%	$18.69		
	Bleeding	0.0%	$0.00		
	Infection	13.0%	$239.23		
	Arterial fibrillation	0.0%	$0.00		
**BR**	Anaemia	12.0%	$95.16	$1,178.77	Woyach, 2018 [11]
	Neutropenia	0.0%	$0.00		
	Febrile neutropenia	7.0%	$764.26		
	Thrombocytopenia	0.0%	$0.00		
	Bleeding	0.0%	$0.00		
	Infection	15.0%	$276.04		
	Arterial fibrillation	3.0%	$43.31		
**IBRU**	Anaemia	7.0%	$55.51	$420.46	Burger, 2020 [12]
	Neutropenia	13.0%	$71.93		
	Febrile neutropenia	0.0%	$0.00		
	Thrombocytopenia	0.0%	$0.00		

Treatment	Adverse Events	%	Weighted Average Cost	Total Cost per Patient	Source
	Bleeding	0.0%	$0.00		
	Infection	12.0%	$220.83		
	Arterial fibrillation	5.0%	$72.18		
VO	Anaemia	9.0%	$71.37	$1,128.97	Al-Sawaf, 2020 [13]
	Neutropenia	53.0%	$293.26		
	Febrile neutropenia	5.0%	$545.90		
	Thrombocytopenia	13.0%	$60.74		
	Bleeding	0.0%	$0.00		
	Infection	7.0%	$128.82		
	Arterial fibrillation	2.0%	$28.87		
ACAL	Anaemia	0.0%	$0.00	$104.28	Sharman, 2021 [14]
	Neutropenia	11.2%	$61.97		
	Febrile neutropenia	0.0%	$0.00		
	Thrombocytopenia	0.0%	$0.00		
	Bleeding	2.8%	$26.43		
	Infection	0.0%	$0.00		
	Arterial fibrillation	1.1%	$15.88		
ACAL+O	Anaemia	0.0%	$0.00	$206.07	Sharman, 2021 [14]
	Neutropenia	30.9%	$170.97		
	Febrile neutropenia	0.0%	$0.00		
	Thrombocytopenia	0.0%	$0.00		
	Bleeding	2.8%	$26.43		
	Infection	0.0%	$0.00		
	Arterial fibrillation	0.6%	$8.66		

1L: first-line treatment, ACAL: acalabrutinib, ACAL+O: acalabrutinib in combination with obinutuzumab, AE: adverse event, BR: bendamustine in combination with rituximab, CLB+O: chlorambucil in combination with obinutuzumab, CLB+R: chlorambucil in combination with rituximab, F: fludarabine, FCR: fludarabine, cyclophosphamide, rituximab, FR: fludarabine in combination with rituximab, IBRU: ibrutinib, VO: venetoclax in combination with obinutuzumab.

Table S12. Severe AE Frequencies per Treatment – 2L/3L

Treatment	Adverse Events	%	Weighted Average Cost	Total Average per Patient	Source
F	Anaemia	8.0%	$63.44	$461.58	Niederle, 2013 [15]
	Neutropenia	17.0%	$94.06		
	Febrile neutropenia	0.0%	$0.00		
	Thrombocytopenia	6.0%	$28.04		
	Bleeding	0.0%	$0.00		
	Infection	15.0%	$276.04		
	Arterial fibrillation	0.0%	$0.00		
FCR	Anaemia	12.0%	$95.16	$1,781.13	Robak, 2010 [16]
	Neutropenia	42.0%	$232.39		
	Febrile neutropenia	12.0%	$1,310.16		
	Thrombocytopenia	11.0%	$51.40		
	Bleeding	0.0%	$0.00		
	Infection	5.0%	$92.01		
	Arterial fibrillation	0.0%	$0.00		

Treatment	Adverse Events	%	Weighted Average Cost	Total Average per Patient	Source
**BR**	Anaemia	13.8%	$109.44	$1,566.67	Seymour, 2018 [3]
	Neutropenia	38.8%	$214.69		
	Febrile neutropenia	9.6%	$1,048.13		
	Thrombocytopenia	10.1%	$47.19		
	Bleeding	0.0%	$0.00		
	Infection	8.0%	$147.22		
	Arterial fibrillation	0.0%	$0.00		
**IBRU**	Anaemia	9.0%	$71.37	$823.90	Munir, 2019 [17]
	Neutropenia	25.0%	$138.33		
	Febrile neutropenia	0.0%	$0.00		
	Thrombocytopenia	10.0%	$46.73		
	Bleeding	10.0%	$94.40		
	Infection	21.0%	$386.46		
	Arterial fibrillation	6.0%	$86.62		
**V**	Anaemia	29.0%	$229.98	$2,287.85	Jones, 2018 [18]
	Neutropenia	51.0%	$282.19		
	Febrile neutropenia	13.0%	$1,419.34		
	Thrombocytopenia	29.0%	$135.51		
	Bleeding	0.0%	$0.00		
	Infection	12.0%	$220.83		
	Arterial fibrillation	0.0%	$0.00		
**VR**	Anaemia	10.8%	$85.65	$1,146.64	Seymour, 2018 [3]
	Neutropenia	57.7%	$319.26		
	Febrile neutropenia	3.6%	$393.05		
	Thrombocytopenia	5.7%	$26.63		
	Bleeding	0.0%	$0.00		
	Infection	17.5%	$322.05		
	Arterial fibrillation	0.0%	$0.00		
**ACAL**	Anaemia	11.0%	$87.23	$280.93	Ghia, 2020 [19]
	Neutropenia	15.0%	$83.00		
	Febrile neutropenia	0.0%	$0.00		
	Thrombocytopenia	4.0%	$18.69		
	Bleeding	0.0%	$0.00		
	Infection	5.0%	$92.01		
	Arterial fibrillation	2.0%	$28.87		

2L: second-line treatment, 3L: third-line treatment, ACAL: acalabrutinib, AE: adverse event, BR: bendamustine in combination with rituximab, F: fludarabine, FCR: fludarabine, cyclophosphamide, rituximab, IBRU: ibrutinib, V: venetoclax, VR: venetoclax in combination with rituximab.

References

1. Al-Sawaf, O.; Zhang, C.; Tandon, M.; Sinha, A.; Fink, A.; Robrecht, S.; al, e. Venetoclax plus obinutuzumab versus chlorambucil plus obinutuzumab for previously untreated chronic lymphocytic leukaemia (CLL14): follow-up results from a multicentre, open-label, randomised, phase 3 trial. *Lancet Oncol* **2020**, *21*, 1188-1200.
2. Stilgenbauer, S.; Eichhorst, B.; Schetelig, J.; Coutre, S.; Seymour, J.F.; Munir, T.; Puvvada, S.D.; Wendtner, C.M.; Roberts, A.W.; Jurczak, W.; et al. Venetoclax in relapsed or refractory chronic lymphocytic leukaemia with 17p deletion: a multicentre, open-label, phase 2 study. *Lancet Oncol* **2016**, *17*, 768-778, doi:10.1016/S1470-2045(16)30019-5.
3. Seymour, J.F.; Kipps, T.J.; Eichhorst, B.; Hillmen, P.; D'Rozario, J.; Assouline, S.; Owen, C.; Gerecitano, J.; Robak, T.; De la Serna, J.; et al. Venetoclax-rituximab in relapsed or refractory chronic lymphocytic leukemia. *N Engl J Med* **2018**, *378*, 1107-1120, doi:10.1056/NEJMoa1713976.
4. Ministry of Health and Long Term Care Ontario Health Insurance Plan. Schedule of benefits for laboratory services. **2020**.
5. Government of Canada. Job bank - Wage report. Available online: https://www.jobbank.gc.ca/wagereport/location/geo9219 (accessed on December).
6. Ministry of Health and Long Term Care Ontario Health Insurance Plan. Schedule of benefits - physician services under the health insurance act. **2021**.
7. Canadian Institute for Health Information (CIHI). Patient cost estimator. **2021**.
8. Eichhorst, B.F.; Busch, R.; Stilgenbauer, S.; Stauch, M.; Bergmann, M.A.; Ritgen, M.; Kranzhofer, N.; Rohrberg, R.; Soling, U.; Burkhard, O.; et al. First-line therapy with fludarabine compared with chlorambucil does not result in a major benefit for elderly patients with advanced chronic lymphocytic leukemia. *Blood* **2009**, *114*, 3382-3391, doi:10.1182/blood-2009-02-206185.
9. Byrd, J.C.; Petersonm, B.L.; Morrison, V.A.; Park, K.; Jacobson, R.; Hoke, E.; Vardiman, J.W.; Rai, K.; Schiffer, C.A.; Larson, R.A. Randomized phase 2 study of fludarabine with concurrent versus sequentialtreatment with rituximab in symptomatic, untreated patients with B-cellchronic lymphocytic leukemia: results from Cancer and LeukemiaGroup B 9712 (CALGB 9712). *Blood* **2003**, *101*, 6-14, doi:10.1182/blood-2002-04-1258.
10. Goede, V.; Fischer, K.; Busch, R.; Engelke, A.; Eichhorst, B.; Wendtner, C.M.; Chagorova, T.; de la Serna, J.; Dilhuydy, M.S.; Illmer, T.; et al. Obinutuzumab plus chlorambucil in patients with CLL and coexisting conditions. *N Engl J Med* **2014**, *370*, 1101-1110, doi:10.1056/NEJMoa1313984.
11. Woyach, J.A.; Ruppert, A.S.; Heerema, N.A.; Zhao, W.; Booth, A.W.; Ding, W.; Bartlett, N.L.; Brander, D.M.; Barr, P.M.; Rogers, K.A.; et al. Ibrutinib Regimens versus Chemoimmunotherapy in Older Patients with Untreated CLL. *N Engl J Med* **2018**, *379*, 2517-2528, doi:10.1056/NEJMoa1812836.
12. Burger, J.A.; Barr, P.M.; Robak, T.; Owen, C.; Ghia, P.; Tedeschi, A.; Bairey, O.; Hillmen, P.; Coutre, S.E.; Devereux, S.; et al. Long-term efficacy and safety of first-line ibrutinib treatment for patients with CLL/SLL: 5 years of follow-up from the phase 3 RESONATE-2 study. *Leukemia* **2020**, *34*, 787-798, doi:https://doi.org/10.1038/s41375-019-0602-x.
13. Al-Sawaf, O.; Zhang, C.; Tandon, M.; Sinha, A.; Fink, A.M.; Robrecht, S.; Samoylova, O.; Liberati, A.M.; Pinilla-Ibarz, J.; Opat, S.; et al. Venetoclax plus obinutuzumab versus chlorambucil plus obinutuzumab for previously untreated chronic lymphocytic leukaemia (CLL14): follow-up results from a multicentre, open-label, randomised, phase 3 trial. *Lancet Oncol* **2020**, *21*, 1188-1200, doi:10.1016/S1470-2045(20)30443-5.
14. Sharman, J.; Egyed, M.; Jurczak, W.; Skarbnik, A.; Pagel, J.; Kamdar, M.; al., e. Acalabrutinib ± obinutuzumab versus obinutuzumab + chlorambucil in treatment-naïve chronic lymphocytic leukemia: Elevate-TN four-year follow up. *J clin Oncol* **2021**, *39*, 7509-7509, doi:10.1016/S0140-6736(20)30262-2.
15. Niederle, N.; Megdenberg, D.; Balleisen, L.; Heit, W.; Knauf, W.; Weiß, J.; Freier, W.; Hinke, A.; Ibach, S.; Eimermacher, H. Bendamustine compared to fludarabine as second-line treatment in chronic lymphocytic leukemia. *Ann Hematol* **2013**, *92*, 653-660, doi:10.1007/s00277-012-1660-6.
16. Robak, T.; Dmoszynska, A.; Solal-Celigny, P.; Warzocha, K.; Loscertales, J.; Catalano, J.; Afanasiev, B.V.; Larratt, L.; Geisler, C.H.; Montillo, M.; et al. Rituximab plus fludarabine and cyclophosphamide prolongs progression-free survival compared with fludarabine and cyclophosphamide alone in previously treated chronic lymphocytic leukemia. *J Clin Oncol* **2010**, *28*, 1756-1765, doi:10.1200/JCO.2009.26.4556.
17. Munir, T.; Brown, J.R.; O'Brien, S.; Barrientos, J.C.; Barr, P.M.; Reddy, N.M.; Coutre, S.; Tam, C.S.; Mulligan, S.P.; Jaeger, U.; et al. Final analysis from RESONATE: Up to six years of follow-up on ibrutinib in patients with previously treated chronic lymphocytic leukemia or small lymphocytic lymphoma. *Am J Hematol* **2019**, *94*, 1353-1363, doi:10.1002/ajh.25638.
18. Jones, J.A.; Mato, A.R.; Wierda, W.G.; Davids, M.S.; Choi, M.; Cheson, B.D.; Furman, R.R.; Lamanna, N.; Barr, P.M.; Zhou, L.; et al. Venetoclax for Chronic Lymphocytic Leukaemia Progressing after Ibrutinib: a Multicentre, Open-Label Phase 2 Trial. *Lancet Oncol* **2018**, *19*, 65-67, doi:10.1016/S1470-2045(17)30909-9.
19. Ghia, P.; Pluta, A.; Wach, M.; Lysak, D.; Kozak, T.; Simkovic, M.; Kaplan, P.; Kraychok, I.; Illes, A.; de la Serna, J.; et al. ASCEND: phase III, randomized trial of acalabrutinib versus idelalisib plus rituximab or bendamustine plus rituximab in relapsed or refractory chronic lymphocytic leukemia. *Am Soc Clin Oncol* **2020**, *38* 2849-2861, doi:10.1200/JCO.19.03355.
